# High tibial osteotomy with individualised alignment and meniscal centralisation improves KOOS sports and recreation and cartilage status compared to conventional Fujisawa‐point alignment without centralisation: A propensity score matching study

**DOI:** 10.1002/jeo2.70594

**Published:** 2025-12-09

**Authors:** Kazushi Horita, Yasutoshi Ikeda, Tomoaki Kamiya, Kodai Hamaoka, Katsunori Takahashi, Yohei Okada, Makoto Emori, Atsushi Teramoto

**Affiliations:** ^1^ Department of Orthopaedic Surgery Sapporo Medical university School of Medicine Sapporo Chuo‐ku Japan

**Keywords:** alignment, centralisation, high tibial osteotomy, joint preserving, medial meniscus extrusion

## Abstract

**Purpose:**

This study was performed to compare clinical outcomes of medial opening‐wedge high tibial osteotomy (MOWHTO) with individualised alignment and medial meniscus centralisation versus conventional alignment targeting the Fujisawa point without centralisation using propensity score matching. It was hypothesised that the individualised approach with centralisation would not be inferior to conventional HTO targeting the Fujisawa point.

**Methods:**

This retrospective matched case–control study analysed 161 consecutive knees treated with MOWHTO. After applying uniform exclusion criteria and 1:1 propensity score matching for demographic, radiographic, and meniscal factors, 24 knees with HTO and centralisation and 24 control knees were compared. The centralisation group received individualised alignment based on patient characteristics, targeting a weight‐bearing line (WBL) ratio of 57.0%–62.5%, whereas the control group followed the standard 62.5% WBL target. The primary outcome was the Knee Injury and Osteoarthritis Outcome Score (KOOS). The secondary outcomes were radiographic alignment and International Cartilage Repair Society (ICRS) cartilage grade on second‐look arthroscopy.

**Results:**

The mean follow‐up duration was 2.5 ± 0.4 years in the centralisation group and 2.5 ± 0.5 years in the control group. Both groups showed significant improvements in all KOOS subscales from preoperative to final follow‐up (all *p* < 0.01). Final KOOS values were comparable, except for a higher Sports and Recreation score in the centralisation group (72.8 ± 21.1 vs. 56.1 ± 27.5; *p* = 0.039). Postoperative alignment was more neutral in the centralisation group (WBL ratio 51.9% ± 8.7% vs. 61.4% ± 7.6%; *p* < 0.001). Improvement in the ICRS grade of the medial femoral condyle was observed in 54.2% of knees in the centralisation group compared with 12.5% in the control group (*p* = 0.001).

**Conclusion:**

MOWHTO with individualised alignment and medial meniscus centralisation achieved clinical outcomes not inferior to conventional alignment targeting the Fujisawa point without centralisation.

**Level of Evidence:**

Level III, retrospective cohort study.

**Abbreviations**
DFO: distal femoral osteotomy
HKA: hip‐knee‐ankle
HTO: high tibial osteotomy
ICRS: international cartilage repair society
JLCA: joint line convergent angle
KL: Kellgren‐Lawrence
KOOS: Knee injury and osteoarthritis outcome scores
MFC: medial femoral condyle
mLDFA: mechanical lateral distal femoral angle
MME: medial meniscus extrusion
MMPRT: medial meniscus posterior root tear
MOWHTO: medial open‐wedge high tibial osteotomy
MPTA: medial proximal tibial angle
MTP: medial tibial plateau
PROMs: patient‐reported outcome measures
SMDs: standardised mean differences
WBL: weight‐bearing line

## INTRODUCTION

High tibial osteotomy (HTO) is a standard procedure for symptomatic medial compartment osteoarthritis (OA) with varus malalignment [2, 11, 24, 33, 41]. Varus malalignment and medial meniscus dysfunction, particularly medial meniscus extrusion (MME), are major contributors to OA progression [12, 14, 17, 18, 34, 42]. MME increases tibiofemoral contact pressure and accelerates cartilage degeneration, underscoring the importance of correcting malalignment to preserve meniscal function.

Meniscus centralisation repositions the extruded meniscus toward the tibial margin, restoring hoop tension and reducing MME. Meniscus centralisation alone has been shown to produce favourable outcomes; [3, 8, 10, 28, 39] however, few studies have examined its combination with HTO [26, 47]. Traditionally, HTO has aimed for valgus overcorrection to the Fujisawa point to promote cartilage repair [16]. More recently, the European Society of Sports Traumatology, Knee Surgery and Arthroscopy (ESSKA) consensus has recommended individualised alignment, targeting a weight‐bearing line (WBL) of 50%–65% based on patient age, activity level, and cartilage status [13, 20, 22, 37]. Some evidence suggests that under‐correction combined with cartilage restoration may yield superior outcomes compared with valgus alignment [1, 20]. These cartilage procedures are rarely used in routine HTO, and some degree of valgus remains necessary for untreated cartilage regeneration [15]. Restoration of meniscal function through centralisation may allow a more neutral correction without compromising cartilage health.

The purpose of this study was to compare the Knee Injury and Osteoarthritis Outcome Score (KOOS) and cartilage status between individualised HTO with centralisation and conventional alignment HTO without centralisation using propensity score matching. It was hypothesised that individualised HTO with centralisation would yield KOOS results not inferior to those of conventional alignment and would demonstrate better cartilage status.

## METHODS

### Patients

This retrospective matched case‐control study analysed anonymized clinical routine care data. Approval was obtained from the Institutional Review Board and Ethics Committee of the institution (approval no. 302‐178). The requirement for informed consent was waived because the data were de‐identified, and patients were allowed to opt out. Medial open‐wedge HTO (MOWHTO) was indicated for symptomatic medial compartment OA with tibial varus malalignment in patients able to undergo postoperative rehabilitation. Beginning in April 2020, medial meniscus centralisation was considered when magnetic resonance imaging (MRI) showed MME of ≥3 mm or when MME was <3 mm but accompanied by a posterior root tear or severe mid‐to‐posterior degeneration. Centralisation was performed in 61.5% of MOWHTO cases. MME was defined as the distance between the peripheral meniscal edge and the tibial plateau margin on coronal MRI.

**Figure 1:**
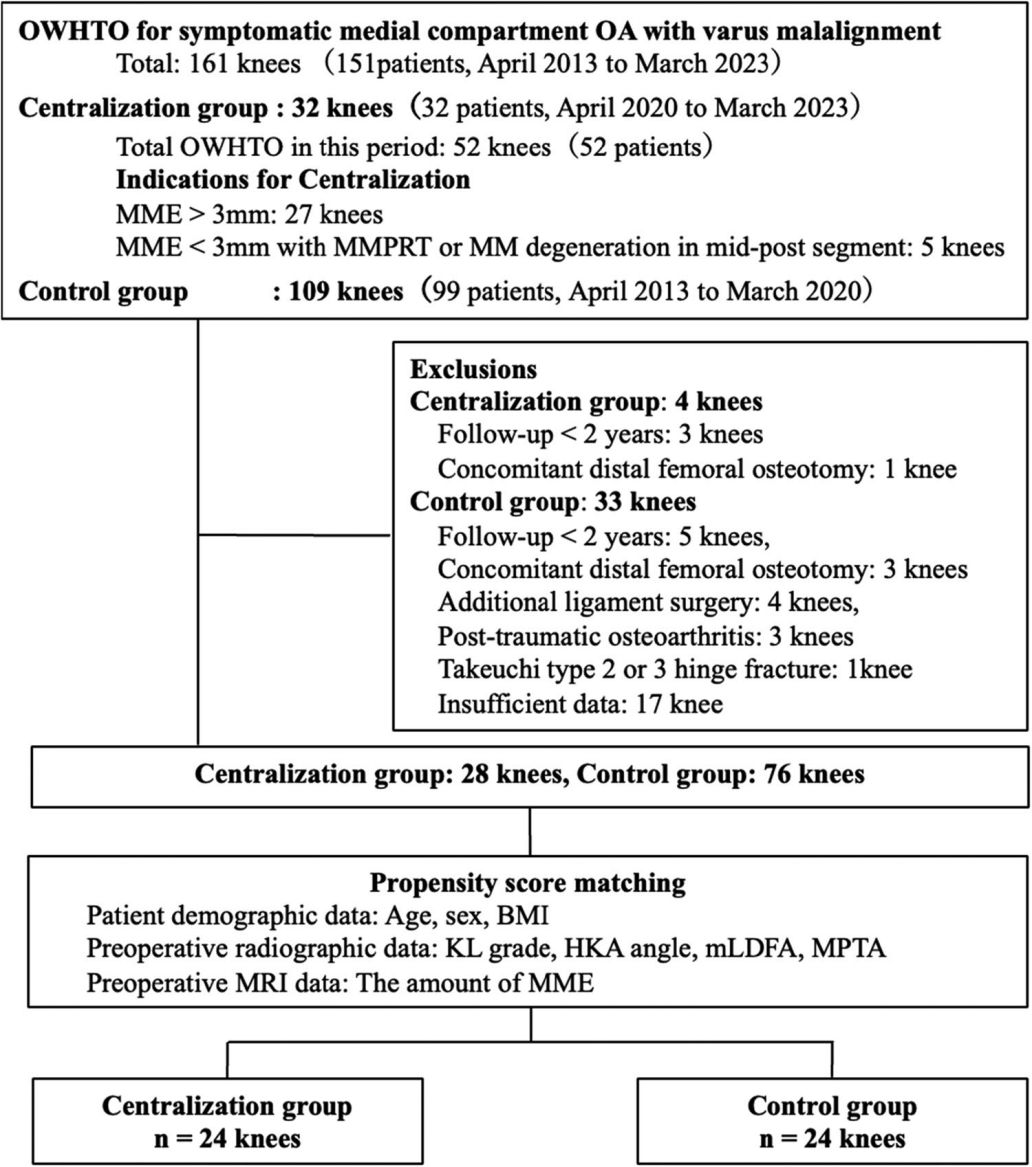
Participant identification and propensity score matching flowchart. BMI, body mass index; HKA, hip–knee–ankle; KL, Kellgren–Lawerence; mLDFA, mechanical lateral distal femoral angle; MM, medial meniscus; MME, medial meniscus extrusion; MMPRT, medial meniscus posterior root tear; MPTA, medial proximal tibial angle; OA, osteoarthritis; OWHTO, open‐wedge high tibial osteotomy.

Between April 2013 and March 2023, 161 knees underwent MOWHTO at the institution. After applying the following exclusion criteria—follow‐up of <2 years, concurrent distal femoral osteotomy, combined ligament surgery, post‐traumatic OA, Takeuchi type 2 or 3 hinge fracture [45], or insufficient data—28 centralisation knees (from 2020–2023) and 76 control knees (from 2013–2020) were eligible for analysis. Propensity score matching was performed based on age, sex, body mass index (BMI), Kellgren–Lawrence (KL) grade, hip–knee–ankle (HKA) angle, mechanical lateral distal femoral angle (mLDFA), medial proximal tibial angle (MPTA), and MME. Patients in the centralisation group (MOWHTO with centralisation; *n *= 24) were matched 1:1 with those in the control group (MOWHTO without centralisation; *n* = 24) (Figure 1).

### Surgical procedures

All procedures were performed by two senior surgeons (Y.I. and T.K.) following standardised protocols. Preoperative planning was conducted using full‐length, weight‐bearing radiographs and the Miniaci method [35]. In the control group, correction was targeted to a WBL ratio of 62.5% (Fujisawa point). In the centralisation group, individualised alignment was determined based on OA severity, age, BMI, and activity level, with a target WBL ratio of 57%–62.5%. For knees with advanced OA (KL grade >3), the target was approximately 62.5%. In younger patients with low BMI and high activity levels, the target was approximately 57% [13, 20, 22, 37]. Exact cut‐off values for age, BMI, or activity level were not defined; these factors were instead used to guide alignment decisions.

Diagnostic arthroscopy was performed before osteotomy to evaluate and manage intra‐articular lesions. Degenerative medial meniscus tears were left untreated when no mechanical symptoms (catching or locking) were present; flap tears were resected, and repairable meniscal tears were sutured.

MOWHTO was performed as previously described [44]. A medial oblique incision was made to expose the proximal tibia. Under fluoroscopic guidance, a biplanar osteotomy was created with a 1‐cm lateral cortical hinge. The osteotomy gap was gradually opened and filled with either hydroxyapatite (BONISH; DePuy Synthes, Tokyo, Japan, and NGK Spark Plug Co. Ltd., Aichi, Japan) or β‐tricalcium phosphate (OSferion; OSferion Biomaterials Corp., Tokyo, Japan). Fixation was achieved using either the TomoFix Osteotomy System (DePuy Synthes) or the TriS Medial HTO Plate System (Olympus Terumo Biomaterial, Tokyo, Japan). Intraoperative fluoroscopic confirmation of the mechanical axis was obtained using an alignment rod to ensure that the WBL passed between the centre of the intercondylar eminence and the lateral intercondylar eminence, remaining above 50%, particularly when under‐correction was intended.

**Figure 2:**
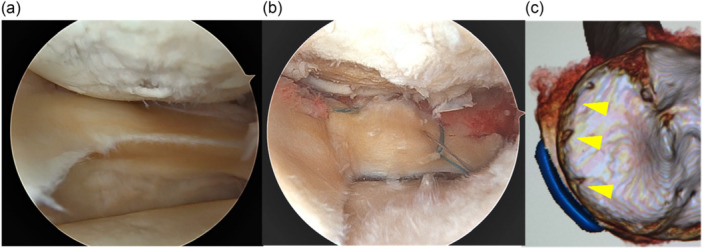
Intraoperative arthroscopy and postoperative three‐dimensional computed tomography. (a) Pre‐centralisation arthroscopic view. (b) Post‐centralisation arthroscopic view. (c) Three‐dimensional computed tomography showing anchor positions (yellow arrowheads).

Meniscus centralisation was performed according to the technique described by Koga et al. [28]. Two to three knotless soft anchors (Knotless FiberTak™; Arthrex, Naples, FL, USA) were inserted from the midbody to the posterior horn of the medial meniscus. Sutures were passed from inferior to superior through the meniscal rim using a suture relay technique, and medialization was achieved by tensioning the sutures through a shuttling loop. In cases involving medial meniscus posterior root tears (MMPRT), additional pull‐out repair was performed using a tibial tunnel and suture tape in a racking hitch knot configuration. Figure 2 shows intraoperative arthroscopic and postoperative three‐dimensional computed tomography images before and after centralisation. All implants were removed approximately 1 year postoperatively.

### Rehabilitation protocol

In both groups, quadriceps strengthening and range of motion (ROM) exercises were initiated immediately after surgery. Partial weight bearing (50%) was permitted at 1–2 weeks and full weight bearing at 2–4 weeks postoperatively. In the centralisation group, ROM was restricted to 90° until week 1, with no limitations thereafter. For patients with MMPRT, this restriction was maintained for 2–4 weeks. The control group had no ROM limitations.

### Primary outcome measure: KOOS

The KOOS was used as the primary outcome measure to evaluate clinical outcomes preoperatively and at the final follow‐up [36]. All assessments were conducted by an investigator who was not involved in the surgeries. Postoperative KOOS evaluations were performed at the same time points in both the centralisation and control groups, with the final assessment conducted at 2–3 years.

### Secondary outcome measures

#### Radiography

**Figure 3:**
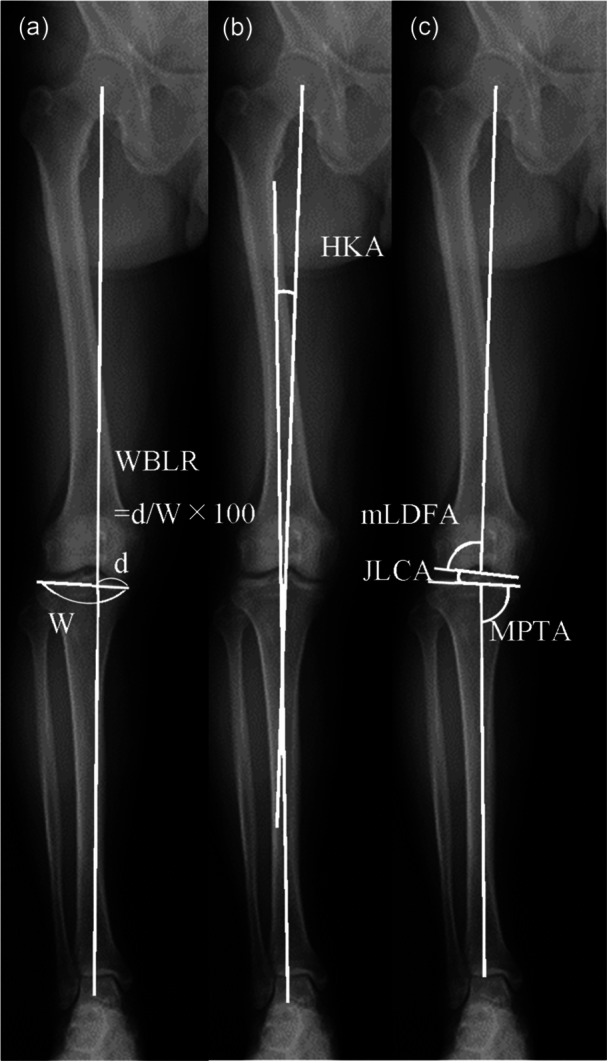
Radiographic measurements on whole‐leg weight‐bearing images. (a) Weight‐bearing line (WBL) ratio: horizontal distance from the WBL to the medial edge divided by the tibial width. (b) Hip–knee–ankle (HKA) angle: angle between the mechanical femoral and tibial axes (varus negative). (c) Medial proximal tibial angle (MPTA): medial angle between the mechanical tibial axis and tibial joint line. Mechanical lateral distal femoral angle (mLDFA): lateral angle between the mechanical femoral axis and femoral joint line. Joint line convergence angle (JLCA): angle between tangents to the femoral condyles and tibial plateau (positive values indicate lateral joint opening).

Coronal plane, whole‐leg, weight‐bearing radiographs were obtained preoperatively and at the final follow‐up, with patients standing bipedally and the patella facing forward. The WBL ratio, HKA angle, mLDFA, MPTA, and joint line convergence angle (JLCA) were measured as described by Paley [40] (Figure 3).

The KL grade was determined using standard standing anteroposterior knee radiographs taken at 3 months postoperatively, 1 year postoperatively, and annually thereafter. Acceptable postoperative alignment was defined as a WBL ratio of 50%–70%, referencing previous reports on Fujisawa‐based (60%–70%) and individualised under‐correction (50%–60%) approaches [16, 20, 51]. The proportion of knees with a postoperative WBL ratio outside this range was also evaluated.

#### Arthroscopy

Cartilage status of the medial femoral condyle (MFC) and medial tibial plateau (MTP) was evaluated using the International Cartilage Repair Society (ICRS) grading system [23, 29]. The highest ICRS grade at each site was recorded based on findings from the initial and second‐look arthroscopies [46].

#### MRI

MME was evaluated using 1.5 T or 3 T MRI scanners (Siemens Healthineers, Erlangen, Germany), as previously described [12]. In the coronal T1‐weighted sequence, the slice showing the largest medial tibial eminence was selected. MME was defined as the distance between two parallel vertical lines: the first drawn at the junction of the horizontal and vertical portions of the MTP, and the second tangential to the medial border of the medial meniscus. MRIs were obtained preoperatively in both groups and postoperatively at 3 months and 1–3 years in the centralisation group.

### Statistical analysis

Propensity scores were generated through logistic regression using age, sex, and BMI, as well as preoperative KL grade, HKA angle, mLDFA, MPTA, and MME, with a 1:1 nearest‐neighbour matching algorithm (greedy method). Model performance was evaluated using the C‐statistic for discrimination and the Hosmer–Lemeshow test for calibration. Covariate balance before and after matching was assessed using standardised mean differences.

An a priori power analysis was performed using G*Power 3.1 version 3.1.9.2 (Franz Faul, Universität Kiel). Based on previous JLCA data (mean difference: 0.5°, pooled standard deviation: 0.55°, effect size *d* = 0.91), 24 knees per group provided >80% statistical power (*α* = 0.05, two‐tailed).

Data normality was assessed using the Kolmogorov–Smirnov test. Continuous variables were compared between groups using independent t‐tests for normally distributed data or Mann–Whitney *U* tests for non‐normally distributed data. Paired comparisons within groups between preoperative and postoperative values were analysed using paired t‐tests or Wilcoxon signed‐rank tests. Categorical variables were analysed using Fisher's exact test. For each knee, the change in cartilage grade between initial and second‐look evaluations was calculated. Improvement was defined as a decrease in ICRS grade, progression as an increase, and stationary as no change. A *p*‐value of <0.05 was considered statistically significant.

All statistical analyses were performed using EZR (Easy R; Saitama Medical Centre, Jichi Medical University, Saitama, Japan) [25]. To assess reliability, radiologic parameters for 30 randomly selected knees were measured twice by two independent orthopaedic surgeons (K.H. and K.H.) at a 2‐week interval, and intra‐ and inter‐observer intraclass correlation coefficients were calculated.

## RESULTS

The C‐statistic (0.72) and Hosmer–Lemeshow test (*p* = 0.976) indicated good discrimination and calibration of the propensity score model. Baseline characteristics, radiographic parameters, and preoperative MME were well balanced between groups, with standardised mean differences of <0.2 (Table 1). Preoperative KOOS values were comparable between the two groups. The mean follow‐up period (from surgery to final evaluation) was 2.5 ± 0.4 years in the centralisation group and 2.4 ± 0.5 years in the control group. The mean correction angle was significantly smaller in the centralisation group than in the control group (8.9° ± 2.9° vs. 7.5° ± 1.2°, *p* = 0.043). Intra‐ and inter‐observer intraclass correlation coefficients were 0.94–0.98 and 0.84–0.96, respectively, indicating high measurement reliability.

**Table 1:** Patient characteristics before and after propensity score matching.

	Total cases (*n* = 104 knees)				Matched cases (*n* = 48 knees)			
	Control group *n* = 76 knees	Centralisation group *n* = 28 knees	SMD	*p* value	Control group *n* = 24 knees	Centralisation group *n* = 24 knees	SMD	*p* value
Age, y	62.9 ± 8.4	63.5 ± 7.5	0.076	0.737	62.0 ± 7.7	63.4 ± 8.1	0.180	0.536
Sex, male/female, knees	27/49	13/15	0.223	0.432	10/14	11/13	0.084	0.999
BMI	26.5 ± 4.2	26.4 ± 3.6	0.034	0.882	26.1 ± 5.2	26.4 ± 3.8	0.068	0.816
Correction angle, °	9.1 ± 2.3	7.6 ± 1.3	0.839	0.001	8.9 ± 2.9	7.5 ± 1.2	0.601	0.043
KL grade, 1/2/3/4, knees	26/39/9/1	10/15/3/0	0.171	0.936	7/14/3/0	10/11/3/0	0.190	0.726
WBL ratio, %	25.6 ± 13.8	30.2 ± 8.1	0.404	0.101	28.2 ± 14.1	29.8 ± 7.7	0.139	0.632
HKA angle, °	−5.1 ± 3.4	−4.2 ± 2.1	0.322	0.190	−4.4 ± 3.6	−4.2 ± 2.0	0.069	0.687*
mLDFA, °	88.6 ± 2.3	87.4 ± 1.8	0.591	0.012	87.5 ± 2.1	87.7 ± 1.5	0.117	0.688
MPTA, °	85.4 ± 2.6	84.8 ± 2.1	0.255	0.274	85.3 ± 2.3	85.2 ± 1.8	0.028	0.923
JLCA, °	2.08 ± 2.27	1.71 ± 1.29	0.203	0.411	1.90 ± 2.86	1.73 ± 1.39	0.016	0.793
MME, mm	3.9 ± 1.7	4.4 ± 1.7	0.243	0.273	4.4 ± 2.1	4.1 ± 1.5	0.123	0.672
MMPRT, knees	14	5	0.015	0.999	4	5	0.099	0.999

*Note*: Matching was based on age and BMI, as well as preoperative KL grade, HKA angle, mLDFA, MPTA, and MME. Values are reported as mean ± standard deviation.

Abbreviations: BMI, body mass index; HKA, hip–knee–ankle; JLCA, joint line convergence angle; KL, Kellgren–Lawrence; mLDFA, mechanical lateral distal femoral angle; MME, medial meniscus extrusion; MMPRT, medial meniscus posterior root tear; MPTA, medial proximal tibial angle; SMD, standardised mean difference; WBL, weight‐bearing line.

* Mann–Whitney *U* test.

### Clinical outcomes

Preoperatively, KOOS subscale scores were comparable between the centralisation and control groups: Symptoms, 61.3 ± 15.8 versus 62.6 ± 15.5; Pain, 69.3 ± 19.1 versus 62.6 ± 22.4; Activities of Daily Living, 68.6 ± 15.6 versus 69.3 ± 18.1; Sports and Recreation, 33.6 ± 18.1 versus 33.1 ± 25.1; and Quality of Life, 32.1 ± 16.0 versus 32.7 ± 20.7 (Table 2).

**Table 2:** Preoperative and final follow‐up KOOS subscale scores in the control and centralisation groups after propensity score matching.

KOOS	Control group (*n* = 24 knees)	Centralisation group (*n* = 24 knees)	*p* value
Symptoms			
Preoperative	62.6 ± 15.5	61.3 ± 15.8	0.817
Last follow‐up	81.1 ± 13.3	80.5 ± 16.2	0.999*
*p*‐Value	**0.0011** *	**0.0021** *	
Pain			
Preoperative	62.6 ± 22.4	69.3 ± 19.1	0.499
Last follow‐up	80.0 ± 17.1	81.0 ± 17.8	0.755*
*p*‐Value	**<0.001** *	**0.00252** *	
Activities of Daily Living			
Preoperative	69.3 ± 18.1	68.6 ± 15.6	0.911
Last follow‐up	84.9 ± 15.5	86.9 ± 12.7	0.735*
*p*‐Value	**<0.001** *	**0.00168** *	
Sports and Recreation			
Preoperative	33.1 ± 25.1	33.6 ± 18.1	0.944
Last follow‐up	56.1 ± 27.5	72.8 ± 21.1	**0.039** *
*p*‐Value	**0.005**	**<0.001**	
Quality of Life			
Preoperative	32.7 ± 20.7	32.1 ± 16.0	0.927
Last follow‐up	62.6 ± 22.4	69.4 ± 19.1	0.313
*p*‐Value	**<0.001**	**<0.001**	

*Note*: Values are reported as mean ± standard deviation. The rightmost *p*‐value column indicates between‐group differences (control vs. centralisation) at each time point. The p‐values in the final row for each parameter indicate within‐group changes from the preoperative to the final follow‐up assessment.

Abbreviation: KOOS, Knee Injury and Osteoarthritis Outcome Score.

* Mann–Whitney *U* test. Bold indicates statistically significant differences (*p* < 0.05).

Both groups showed significant improvements compared with their preoperative values across all KOOS subscales from baseline to final follow‐up (all *p* < 0.01).

At the final follow‐up, the centralisation and control groups again showed similar scores for Symptoms (80.5 ± 16.2 vs. 81.1 ± 13.3, *p* = 0.999), Pain (81.0 ± 17.8 vs. 80.0 ± 17.1, *p* = 0.755), Activities of Daily Living (86.9 ± 12.7 vs. 84.9 ± 15.5, *p* = 0.735), and Quality of Life (69.4 ± 19.1 vs. 62.6 ± 22.4, *p* = 0.313), whereas the centralisation group achieved a higher Sports and Recreation score (72.8 ± 21.1 vs. 56.1 ± 27.5, *p* = 0.039).

### Radiography

Preoperatively, radiographic alignment parameters showed no significant differences between groups (Table 3). The mean WBL ratio was 28.2% ± 14.1% in the control group and 29.8% ± 7.7% in the centralisation group; the HKA angle was −4.4° ± 3.6° and −4.2° ± 2.0°; and the MPTA was 85.3° ± 2.2° and 85.2° ± 1.8°, respectively. Both groups demonstrated significant postoperative changes from baseline (all *p* < 0.001).

**Table 3:** Preoperative and final follow‐up radiographic parameters in the control and centralisation groups after propensity score matching.

	Control group (*n* = 24 knees)	Centralisation group (*n* = 24 knees)	*p* value
WBL ratio, %			
Preoperative	28.2 ± 14.1	29.8 ± 7.7	0.632
Last follow‐up	61.4 ± 7.6	51.9 ± 8.7	**<0.001**
*p*‐Value	**<0.001**	**<0.001**	
HKA, °			
Preoperative	−4.4 ± 3.6	−4.2 ± 2.0	0.687*
Last follow‐up	3.1 ± 1.6	0.7 ± 2.3	**<0.001**
*p*‐Value	**<0.001**	**<0.001**	
mLDFA, °			
Preoperative	87.5 ± 2.1	87.7 ± 1.5	0.688
Last follow‐up	87.5 ± 1.7	87.6 ± 2.2	0.261
*p*‐Value	0.984	0.556	
MPTA, °			
Preoperative	85.3 ± 2.2	85.2 ± 1.8	0.923
Last follow‐up	92.6 ± 1.9	90.7 ± 2.0	**0.003**
*p*‐Value	**<0.001**	**<0.001**	
JLCA, °			
Preoperative	1.9 ± 2.9	1.7 ± 1.4	0.718*
Last follow‐up	2.6 ± 2.2	2.0 ± 1.4	0.488*
*p*‐Value	0.301	0.308	

*Note*: Values are reported as mean ± standard deviation. The rightmost *p*‐value column indicates between‐group differences (control vs. centralisation) at each time point. The *p*‐values in the final row for each parameter indicate within‐group changes from the preoperative to the final follow‐up assessment.

Abbreviations: JLCA, joint line convergence angle; HKA, hip–knee–ankle; mLDFA, mechanical lateral distal femoral angle; MPTA, medial proximal tibial angle; WBL, weight‐bearing line.

* Mann–Whitney *U* test. Bold indicates statistically significant differences (*p* < 0.05).

At the final follow‐up, the control group improved to a WBL ratio of 61.4% ± 7.6%, HKA angle of 3.1° ± 1.6°, and MPTA of 92.6° ± 1.9°, while the centralisation group improved to 51.9% ± 8.7%, 0.7° ± 2.3°, and 90.7° ± 2.0°, respectively (all *p* < 0.001 compared with preoperative values). When compared between groups at the final follow‐up, the centralisation group maintained a more neutral alignment, with a significantly lower WBL ratio and HKA angle (both *p* < 0.001) and a lower MPTA (*p* = 0.003), whereas the mLDFA (87.6° ± 2.2° vs. 87.5° ± 1.7°) and JLCA (2.1° ± 1.4° vs. 2.6° ± 2.2°) did not differ significantly.

At 3 months postoperatively, 12.5% (3 of 24 knees) in the centralisation group and 16.7% (4 of 24 knees) in the control group were outside the acceptable range. At the final follow‐up, 16.7% (4 of 24 knees) in the centralisation group and 8.3% (2 of 24 knees) in the control group remained outside this range.

### Arthroscopy

Preoperatively, there were no significant differences between groups in ICRS grades for the MTP (*p* = 0.370) or MFC (*p* = 0.275) (Table 4). On second‐look arthroscopy, improvements in MTP grade were more frequent in the centralisation group (37.5% vs. 8.3%), though this difference did not reach statistical significance (*p* = 0.051). By contrast, the centralisation group showed a significantly higher rate of MFC grade improvement than the control group (54.2% vs. 12.5%; *p* = 0.001). At the time of second‐look arthroscopy, the centralisation group also demonstrated significantly better MFC ICRS grades than the control group (*p* = 0.008).

**Table 4:** ICRS cartilage grades of the control and centralisation groups after propensity score matching.

	Control group (*n* = 24 knees)	Centralisation group (*n* = 24 knees)	*p* value
Preoperative 0/1/2/3/4, knees			
MTP	2/5/6/4/7	0/6/8/7/3	0.370
MFC	2/0/5/10/7	1/2/10/6/5	0.275
Postoperative 0/1/2/3/4, knees			
MTP	2/3/7/5/7	0/10/5/5/4	0.141
MFC	2/5/6/4/7	0/6/8/7/3	**0.008**
Change in ICRS grade			
* MTP*			0.0506
Improved knee	2 (8.3)	9 (37.5)	
Stationary knee	16 (66.7)	12 (50.0)	
Progressed knee	6 (25.0)	3 (12.5)	
* MFC*			**0.001**
Improved knee	3 (12.5)	13 (54.2)	
Stationary knee	16 (66.7)	9 (37.5)	
Progressed knee	5 (20.8)	2 (8.3)	

*Note*: Values are reported as counts and percentages. Bold indicates statistically significant differences (*p* < 0.05).

Abbreviations: ICRS, International Cartilage Regeneration and Joint Preservation Society; MFC, medial femoral condyle; MTP, medial tibial plateau.

### MRI

**Figure 4:**
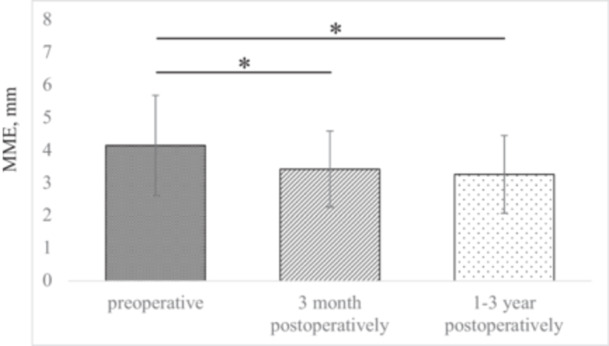
MME pre‐ and post‐MOWHTO in the centralisation group. Asterisks (*) indicate significant differences; error bars represent standard deviations. Final follow‐up was conducted at 1–3 years (mean, 2.0 ± 0.6 years). Postoperative magnetic resonance imaging data were not available for the control group. MME, medial meniscus extrusion; MOWHTO, medial opening‐wedge high tibial osteotomy.

In the centralisation group, mean MME was significantly reduced compared with preoperative values—by 0.72 mm at 3 months postoperatively (3.4 ± 1.2 vs. 4.1 ± 1.5 mm, *p* = 0.003) and by 0.84 mm at 1–3 years postoperatively (3.2 ± 1.2 vs. 4.1 ± 1.5 mm, *p* = 0.003) (Figure 4). However, no significant difference was observed between the 3‐month and 1‐ to 3‐year postoperative evaluations (3.4 ± 1.2 vs. 3.2 ± 1.5 mm, *p* = 0.944; mean follow‐up, 2.0 ± 0.6 years).

## DISCUSSION

HTO with meniscal centralisation and individualised alignment improved KOOS values comparably to conventional HTO targeting the Fujisawa point. Both the centralisation and control groups showed significant within‐group improvements and similar outcomes at approximately 2.5 years (minimum, 2 years). With centralisation, postoperative alignment was closer to neutral, while clinical outcomes remained equivalent to those of the control group. However, because meniscal centralisation and alignment correction were performed concurrently, their individual contributions to these outcomes could not be determined, and postoperative MME was evaluated only in the centralisation group.

Meniscal centralisation has been shown to effectively restore meniscal function in cases of extrusion or degeneration [8, 9, 10, 28, 30, 50]. Ozeki et al. [39] demonstrated delayed cartilage degeneration after centralisation in a rat model. However, most clinical reports have not examined this procedure in the context of varus leg malalignment. Because varus lower‐limb alignment is a known risk factor for MME, the degree of alignment should be considered when planning centralisation [18, 48]. Cadaveric studies indicate that centralisation can temporarily restore meniscal function, though repeated loading may lead to re‐extrusion [4]. A large preoperative MME has also been associated with persistent pain and recurrent varus following HTO in clinical settings [43, 49]. Several investigations of MME changes in MMPRT have reported limited reduction with either HTO or meniscal root repair alone [5, 7, 21, 31, 32], whereas others have shown that MMPRT repair combined with centralisation reduces MME by approximately 1.5 mm at 1 year in KL grade 2 knees [50]. In the present study, MOWHTO combined with centralisation significantly reduced MME immediately postoperatively, although the long‐term decrease was modest (0.72 mm at 3 months and 0.84 mm at 1–3 years), likely reflecting the inclusion of more advanced KL grade 2–3 knees. Collectively, these findings suggest that the efficacy of meniscal centralisation depends on baseline OA severity and meniscal condition, and that alignment correction remains an essential adjunct in patients with lower‐limb malalignment.

The Fujisawa point is considered the standard target alignment after HTO, although the original study evaluated cartilage regeneration in advanced OA by second‐look arthroscopy without addressing meniscal procedures [16]. The ESSKA recommends individualised alignment based on OA severity, age, BMI, and activity level [13, 20, 22, 37], and several reports have suggested that neutral alignment may be preferable when combined with cartilage repair [1, 6, 20]. A neutral WBL ratio (55%) after HTO with autologous chondrocyte implantation has been shown to achieve the highest graft survival, emphasising the importance of appropriate mechanical loading [1]. Similarly, improved cartilage quality with near‐neutral alignment has been demonstrated on T2 mapping [6]. Nevertheless, a systematic review found no clear clinical benefit of HTO combined with cartilage procedures over HTO alone, indicating that when cartilage regeneration depends solely on alignment, adequate valgus correction remains necessary [15]. The present study aligns with the ESSKA concept of individualised alignment. By combining meniscal centralisation, meniscal function was reconstructed, and signs of medial cartilage improvement were observed without direct cartilage repair. Restoring meniscal function may help create an optimal loading environment, suggesting that cartilage healing can be supported without the need for excessive valgus correction.

Neutral alignment is considered beneficial for patients seeking high functional recovery, such as those wishing to resume sports activities; however, concerns regarding persistent pain and recurrent varus remain [19, 27, 38]. In the present study, individualised alignment combined with meniscal centralisation achieved short‐term outcomes comparable to conventional valgus correction and showed significant improvement only in the KOOS Sports and Recreation subscale. Neutral alignment likely contributed to higher functional scores, while centralisation may have provided additional benefits for pain relief and cartilage protection, although the current data cannot confirm these effects. In comparison, HTO with a neutral target alignment, with or without meniscal centralisation, has been reported to produce similar clinical outcomes [26]. However, the centralisation group demonstrated medial joint space widening (1.9–2.7 mm) and a decrease in JLCA (4.2°–3.3°), suggesting cartilage regeneration. In the present study, the centralisation group also achieved a more neutral alignment and higher KOOS Sports and Recreation scores (72.8 vs. 56.1), along with a greater improvement in MFC ICRS grade, indicating potential cartilage protection. Although no significant JLCA reduction was observed—likely because preoperative JLCA values were already low—the final mean JLCA remained within a clinically acceptable range (≤2°). Overall, HTO with meniscal centralisation under individualised alignment achieved clinical outcomes comparable to those of conventional valgus correction while potentially contributing to cartilage preservation. Long‐term studies are warranted to determine whether meniscal centralisation provides sustained benefits in terms of pain, function, and cartilage status.

This study has several limitations. Individualised alignment and centralisation were performed concurrently, making it impossible to distinguish their respective effects. Target alignment was determined at the surgeon's discretion without predefined cut‐off values for age, BMI, or activity level. The sample size was relatively small, and the retrospective design limited control over potential confounders. The mean follow‐up duration (2.5 years) was also relatively short. Finally, MME was assessed only in the centralisation group, limiting direct comparison. Nonetheless, this is the first study to compare individualised alignment HTO with centralisation against traditional alignment HTO without centralisation using propensity score matching, and the findings suggest comparable clinical outcomes.

## CONCLUSION

MOWHTO with individualised alignment and medial meniscus centralisation achieved clinical outcomes that were not inferior to those of conventional alignment targeting the Fujisawa point without centralisation and may provide additional benefits for cartilage preservation and sports‐related function.

## AUTHOR CONTRIBUTIONS

Yasutoshi Ikeda and Kazushi Horita were involved in study design. Kazushi Horita and Kodai Hamaoka contributied to data collection. Kazushi Horita performed the statistical analysis and drafted the manuscript. Tomoaki Kamiya, Kodai Hamaoka, Katsunori Takahashi, Yohei Okada, Makoto Emori, and Atushi Teramoto critically revised the report, commented on drafts of the manuscript. All authors approved the final report.

## CONFLICT OF INTEREST STATEMENT

The authors declare no conflicts of interest.

## ETHICS STATEMENT

The study protocol was approved by the Ethics committee of Sapporo Medical University Hospital (IRB Number. 302‐178). All patients provided informed consent for the surgical procedure.

## Data Availability

Data are available from the corresponding author upon reasonable request.
